# Dietary Vitamin E as a Protective Factor for Parkinson's Disease: Clinical and Experimental Evidence

**DOI:** 10.3389/fneur.2019.00148

**Published:** 2019-02-26

**Authors:** Tommaso Schirinzi, Giuseppina Martella, Paola Imbriani, Giulia Di Lazzaro, Donatella Franco, Vito Luigi Colona, Mohammad Alwardat, Paola Sinibaldi Salimei, Nicola Biagio Mercuri, Mariangela Pierantozzi, Antonio Pisani

**Affiliations:** ^1^Department of Systems Medicine, University of Rome Tor Vergata, Rome, Italy; ^2^IRCCS Fondazione Santa Lucia, Rome, Italy; ^3^Department of Biomedicine and Prevention, University of Rome Tor Vergata, Rome, Italy

**Keywords:** Parkinson's disease, Vitamin E, antioxidant, neuroprotection, protective factors, diet, PINK1, synaptic plasticity

## Abstract

Effective disease-modifying treatments are an urgent need for Parkinson's disease (PD). A putative successful strategy is to counteract oxidative stress, not only with synthetic compounds, but also with natural agents or dietary choices. Vitamin E, in particular, is a powerful antioxidant, commonly found in vegetables and other components of the diet. In this work, we performed a questionnaire based case-control study on 100 PD patients and 100 healthy controls. The analysis showed that a higher dietary intake of Vitamin E was inversely associated with PD occurrence independently from age and gender (OR = 1.022; 95% CI = 0.999–1.045; *p* < 0.05), though unrelated to clinical severity. Then, in order to provide a mechanistic explanation for such observation, we tested the effects of Vitamin E and other alimentary antioxidants *in vitro*, by utilizing the homozygous PTEN-induced kinase 1 knockout (*PINK1*^−/−^) mouse model of PD. *PINK1*^−/−^ mice exhibit peculiar alterations of synaptic plasticity at corticostriatal synapses, consisting in the loss of both long-term potentiation (LTP) and long-term depression (LTD), in the absence of overt neurodegeneration. Chronic administration of Vitamin E (alpha-tocopherol and the water-soluble analog trolox) fully restored corticostriatal synaptic plasticity in *PINK1*^−/−^ mice, suggestive of a specific protective action. Vitamin E might indeed compensate PINK1 haploinsufficiency and mitochondrial impairment, reverting some central steps of the pathogenic process. Altogether, both clinical and experimental findings suggest that Vitamin E could be a potential, useful agent for PD patients. These data, although preliminary, may encourage future confirmatory trials.

## Introduction

Parkinson's disease (PD) is a common neurodegenerative disorder, idiopathic, and multifactorial, mainly due to the loss of dopaminergic neurons in the *Substantia Nigra pars compacta* (SNpc) and to excessive brain accumulation of α-synuclein positive cytoplasmic Lewy bodies (LB). PD causes a progressive and disabling syndrome, including motor and non-motor disturbances, which severely impair patients' quality of life. Therefore, effective disease modifying treatments represent an unmet clinical need (1–3).

Successful neuroprotection may imply a combined approach against the different partners of neurodegeneration (4). Since oxidative stress is a major player of the neurodegenerative process in PD, the modulation of redox balance has been extensively explored as a potential strategy to prevent neural death and disease progression. Indeed, different antioxidant agents are under investigation in several clinical trials (5–9). Besides synthetic compounds, an invaluable source of natural antioxidants is food, namely fruits and vegetables (10). Therefore, an antioxidant-rich diet could represent a viable option to boost antioxidant pathways counteracting neurodegeneration.

Multiple antioxidant species can be found in food, which operate differently in cellular metabolism. Vitamin E, in particular, is a powerful antioxidant, found in plants and abundant in many aliments consumed in our diet (11). Vitamin E family includes a number of lipophilic molecules (α-, β-, γ-, δ-tocopherols and α-, β-, γ-, δ-tocotrienols), whose antioxidant properties rely on lipoperoxyl radical scavenging activity (11). Their neuroprotective effects have been demonstrated in multiple experimental models; likewise, the reduced levels of these molecules in humans have been associated with the occurrence of neurodegenerative diseases (12, 13).

We hypothesized that a higher dietary intake of Vitamin E might protect from progressive neurodegeneration in PD. Therefore, we conducted a study including: (1) a retrospective assessment of dietary Vitamin E intake (VEI) in PD patients compared to healthy controls, aimed at determining if a different dietary VEI is associated with diverse clinical conditions; (2) an *in vitro* protocol in brain slices of a PD mouse model, aimed at evaluating the effects of Vitamin E on synaptic plasticity abnormalities, a peculiar endophenotype observed in distinct PD models. Specifically, we used homozygous PTEN-induced kinase 1 (*PINK1*) knockout mice (*PINK1*^−/−^), an established model of subclinical PD, which might reflect the early phases of the disease. In this model, we previously observed a significant decrease in dopamine release, which is the major determinant of the loss of bidirectional synaptic plasticity at corticostriatal synapses. Indeed, both long-term potentiation (LTP) and long-term depression (LTD) are impaired in these mice, in the absence of overt neuronal degeneration, thereby representing an early pathophysiological event preceding cellular death (14–16).

## Methods

### Case-Control Study

#### Population

The study involved 200 consecutive subjects (100 PD patients and 100 sex/age matched controls), afferent to the Neurology Unit of Tor Vergata University Hospital (Rome, Italy). PD was diagnosed according to the United Kingdom PD Society Brain Bank criteria. Controls (CTL) were healthy subjects, without history of neurological diseases or neurological signs at clinical examination, enrolled among non-blood relatives of patients. Exclusion criteria were cognitive decline with Mini-Mental status Examination (MMSE) <25 (adjusted for age and educational level); gastrointestinal disorders and malabsorption; abdominal surgery; diabetes; obesity (BMI > 29); alcoholism; internal failures (e.g., liver, heart); feeding problems; dietary restrictions; habit to taking vitamins integration. All the participants signed a written informed consent. The study was carried out according to the Declaration of Helsinki and was approved by the local ethical committee (Tor Vergata, Rome—Italy; number 98–09).

#### Assessment of VEI

All subjects underwent a structured *ad hoc* interview assessing dietary habits: the interview relied on a questionnaire, including explicative pictures to avoid misunderstanding. Subjects were asked how frequently each specific Vitamin E-rich aliment was habitually consumed in the preceding year (2 = more than once a week; 1 = at least once a month; 0 = never). Vitamin E-rich aliments' daily portions were named in a list including fresh fruits (e.g., kiwi, mango), dried fruits (e.g., almonds, walnuts), vegetable (e.g., spinach, broccoli), seeds (e.g., sunflower, pumpkin), oil (e.g., olive, sunflower), fish (e.g., bluefish, crayfish); (source: US Department of Agriculture, USDA (17)). Individual VEI was finally estimated by summing the products of each food's vitamin E content (mg) ^*^ the frequency of eating (0, 1, 2). Vitamin E content values were obtained from the Swedish Food Administration Database (18).

#### Statistical Analysis

The distribution of collected variables was preliminary examined with the Shapiro–Wilk test. Then, the non-normally distributed data were log-transformed to allow statistical analysis. Differences between the groups were tested by parametric (one-way ANOVA) or non-parametric (chi-square) tests, as appropriate. In addition, possible differences in the VEI depending on the H&Y stage and gender were tested by using the one-way ANOVA. The association between PD and VEI was assessed by means binomial logistic regression, adjusting the model for age and gender.

### Experimental Electrophysiology on PD Mouse Model

#### Animal Model and Experimental Setting

Treatment and handling of animals were carried out in accordance with both the EC and Italian guidelines (86/609/EEC; DLS 116/1992, Directive 2010/63/EU; DLS/26 04/03/2014) and were further approved by the University of Rome Tor Vergata statute (n. 153/2001A) and by Animal Care and Use Committee of University of Rome “Tor Vergata.” Transgenic mice (8- to 10-weeks old) were generated as previously described (14).

Intracellular recordings were obtained from striatal neurons in a parasagittal brain slice (300 μm) (9, 19, 20). A single slice was transferred in a recording chamber (35°C, 2–3 ml/min) and submerged in a continuously flowing Krebs' solution (35°C, 2–3 ml/min) bubbled with 95% O_2_ and 5% CO_2_. Kreb's solution was composed of (in mM): 126 NaCl, 2.5 KCl, 1.3 MgCl_2_, 1.2 NaH_2_PO_4_, 2.4 CaC1_2_, 10 glucose, and 18 NaHCO_3_. Intracellular recording electrodes were filled with 2 M KCl (30–60 MΩ). To evoke excitatory postsynaptic potentials (EPSPs), a bipolar electrode was placed in the white matter, in close proximity to the recording electrode or in layer VI of the cortex. Test stimuli were delivered at a frequency of 0.1 Hz in the presence of 50 μM Picrotoxin to block GABA A-mediated responses. The pharmacological effects on EPSPs recorded from knockout mice (*PINK1*^−/−^) were calculated as percentage of control amplitude in the wild-type (WT) or *PINK1*^+/+^ neuronal population.

For high-frequency stimulation (HFS, three trains 100 Hz, 3 s, 20 s apart), stimulus intensity was raised to reach threshold level. After HFS delivery, the amplitude of EPSPs was plotted over-time as percentage of the control EPSP. Magnesium was omitted from the medium for LTP induction (9, 19, 20).

Signals were recorded with an Axoclamp 2B amplifier (Axon Instruments, Foster City CA 94404, USA), displayed on a oscilloscope and stored on PC using Digidata 1,500 A and pClamp 10.6 (Axon Instruments, Molecular Devices, USA). Data were examined of line by clampfit 10.7 software (Axon Instruments, Molecular Devices, USA). After initial analysis, all data were elaborated by Origin Microcal 2016 (Adalta) software.

#### Treatments

Effects of Vitamin E (alpha-tocopherol and the water-soluble analog Trolox) were assessed in comparison to other antioxidant agents of alimentary origin, in order to test their action specificity. In particular, for our experiments we specifically selected: beta-carotene (21, 22), lycopene (22, 23), lutein (24), folic acid (25), ascorbic acid/Vitamin C (26), retinol/Vitamin A (27), Vitamin K1/phylloquinone, and Vitamin K2/menaquinones (28, 29).

The effects of drug treatments in PINK^−/−^ mice were tested in two different conditions: (1) *ex-vivo*, by acute preincubation of parasagittal brain slices; (2) *in vivo*, by chronically administered intraperitoneal injections.

For acute treatment, a single slice was incubated, from 40 min before HFS induction and for the duration of the whole experiment (about 1 h), in a bath solution containing the drug dissolved in Krebs' solution. Selected compounds were used in bath at the respective dose of: alpha-tocopherol = 100 μM and Trolox = 100 μM (9); beta-carotene = 100 μM (30); lutein = 20 μM (24); lycopene = 5 μM (22, 23); folic acid = 100 μM (25); Vitamin A = 1 μM (27); Vitamin C = 1–3 mM (26); Vitamin K1 = 20 μM, and Vitamin K2 = 10 μM (28, 29).

For chronic treatments, all compounds were solved in ringer lactate and administered via intraperitoneal injections for 7 days consecutively (9). Dose treatment was: alpha-tochoperol = 100 mg/kg/7 days and Trolox = 5 mg/kg/7 days (9); beta-carotene = 2 mg/kg/7 days (31); lutein = 3 mg/kg/7 days (32); lycopene = 50 mg /kg/7 days (31); folic acid = 2 mg/kg/7 days (33); Vitamin A = 0.5 mg/kg/7 days (31); Vitamin C = 100 mg/kg/7 days (34); Vitamin K2 = 50 mg/kg/7 days (35); Vitamin K1 = 150 mg/kg/7 days (36).

#### Drug Source

Beta-carotene, lycopene, folic acid, lutein, folic acid, Vitamin A, Vitamin C, Vitamin K1 and K2, alpha-tochopherol, and Trolox were purchased from Sigma-Adrich, Italy. All the other drugs were purchased by Panreac Quimica (Spain).

#### Statistical Analysis

Data are presented as mean ± standard error of the mean (SEM). Statistical significance between pre and post HFS stimulation was evaluated using Student *T*-test. Percentage values were calculated for each individual experiment. An analysis of variance with the Tukey's *post-hoc* test was performed among the groups (*P* < 0.05; alpha = 0.01). Statistical significance was set at <0.05.

## Results

### Case-Control Study

Clinical-demographic parameters and the VEI of the study population are summarized in Table 1. PD and CTL were homogeneous in age and gender distribution; VEI was significantly higher in CTL (38.4 mg ± 17.8) than PD (31.6 mg ± 13.5; Statistical analysis was conducted on Log-transformed values, resulting *p* < 0.05). Conversely, VEI did not differ depending on the gender in both groups, neither among the stages of H&Y in PD patients. The binomial logistic regression showed that VEI was directly associated with CTL status, independently from age and gender (Odd Ratio, OR = 1.022; 95% CI = 0.999–1.045; *p* < 0.05).

**Table 1:** Clinical-demographic parameters and VEI values of the study population.

		**PD**		**CTL**		**Significance**
Gender	(M/F)	55.2%	44.8%	46.9%	53.1%	ns
Age (years)	Mean	63.3		60.5		
	St.dev.	8.5		10.6		
Log10 Age	Mean	1.80		1.77		ns
	St.dev.	0.06		0.08		
VEI (mg)	Mean	31.6		38.4		
	St.dev.	13.5		17.8		
Log10 VEI	Mean	1.45		1.54		*p < 0.05*
	St.dev.	0.21		0.21		
H&Y	Mean	2.4		–		
	St.dev.	0.7		–		

### Electrophysiology in PD Mouse Model

**Figure 1:**
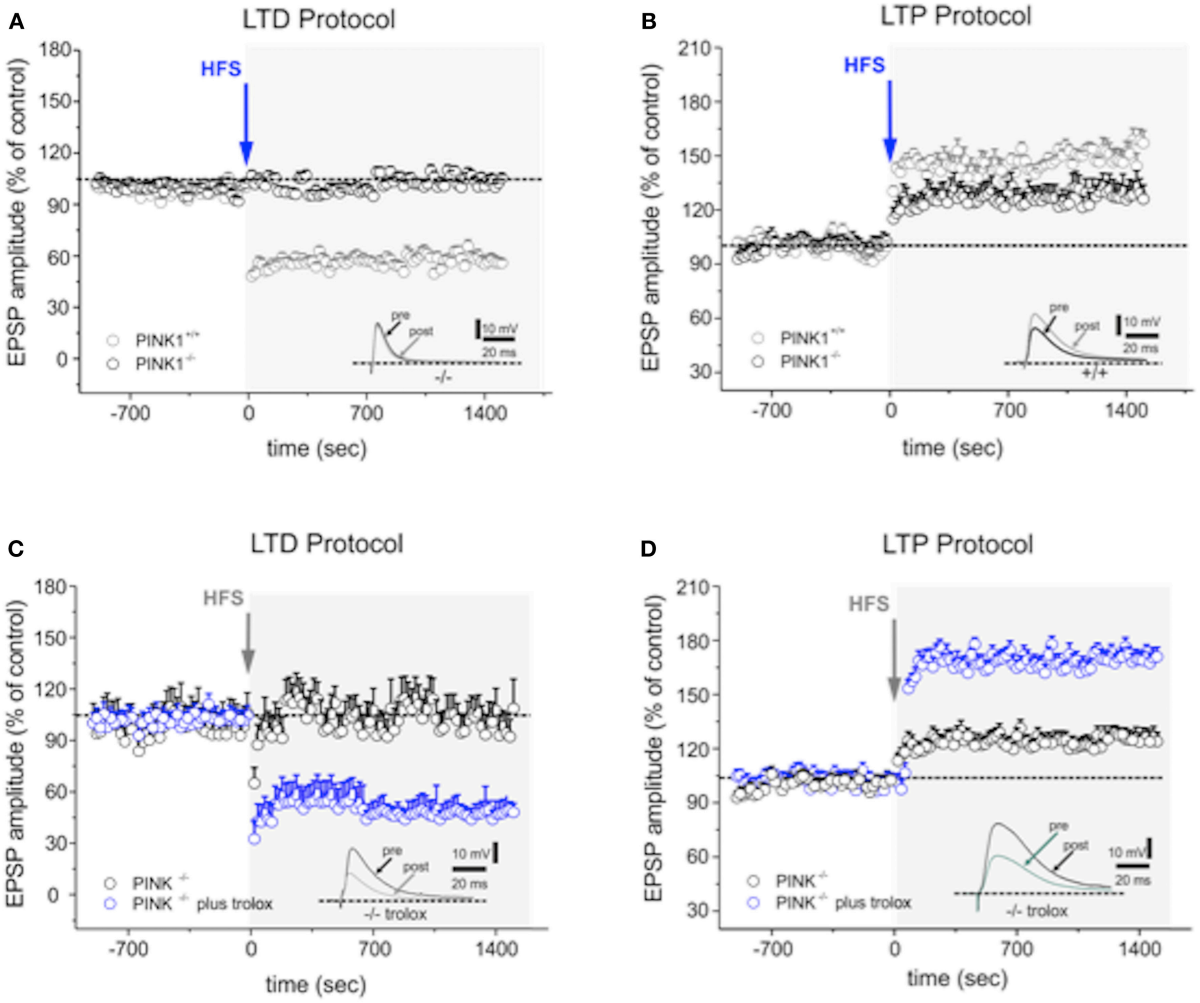
Trolox fully rescues both forms of altered synaptic plasticity in *PINK1*^−/−^ mice. **(A)** Time-course of LTD in WT and *PINK1*^−/−^ mice recorded from parasagittal slices. HFS (arrow) induces LTD in WT mice (gray circles), but not in *PINK1*^−/−^ mice (black circles). The inset shows a representative sample of EPSPs recorded in *PINK1*^−/−^ before (pre) and 20 min after (post) HFS. **(B)** The LTP induction protocol causes LTP in WT mice but not in *PINK1*^−/−^ mice (black circles). The magnitude of LTP measured in *PINK1*^−/−^ mice is significantly reduced. The inset shows a representative sample of EPSPs recorded in *PINK1*^−/−^ before (pre) and 20 min after (post) HFS. **(C,D)** In slices acutely treated with Trolox 100 μM (blue circles) or in slices from mice chronically treated with Trolox, both LTD **(C)** and LTP **(D)** is rescued. LTD is fully rescued, while LTP increases in magnitude as in normal condition. The insets show two representative samples of EPSPs recorded in *PINK1*^−/−^ before (pre) and 20 min after (post) HFS. Each data point represents the mean ± SEM from acute and chronic treatment, respectively.

According to our previous findings (14, 16), HFS protocol performed on parasagittal slice preparation induced a robust LTD in MSNs recorded from WT mice (58.24 ± 3.79% of control; *n* = 16 Figure 1A), whereas it failed to elicit LTD in *PINK1*^−/−^ mice (99.61 ± 2.88% of control; *n* = 22 *p* < 0.05 *t*-test Figure 1A). After removal of magnesium from the bathing medium, HFS induced LTP in WT mice (168.26 ± 5.21% of control; *n* = 12; *t*-test *p* < 0.05 Figure 1B). In *PINK1*^−/−^ mice, HFS also increased EPSPs compared to pre HFS (123.39 ± 4.88% of control; *n* = 12; *t*-test *p* < 0.05, Figure 1B), but the magnitude was significantly lower than WT mice (*p* < 0.05 ANOVA), suggesting the impairment of this form of plasticity.

None of the other drugs, but Vitamin E, was able to rescue either LTD or LTP in both acute and chronic treatment (>5 observations for each experimental condition; *T*-test, *p* > 0.05; Table 2). Specifically, Trolox (the water-soluble analog of Vitamin E) fully rescued both the forms of synaptic plasticity either after acute or chronic administration (LTD protocol: 55.88 ± 6.01% of control; *n* = 16; *t*-test *p* < 0.05 Figure 1C; LTP protocol: 175.30 ± 4.88% of control; *n* = 10; *t*-test *p* < 0.05 Figure 1D, Table 2); alpha-tochoperol restored in chronic conditions (intraperitoneal injection) (LTD protocol: 56.9 ± 4.21% of control; *n* = 8; *t*-test *p* < 0.05; LTP protocol: 177.2 ± 6.36% of control; *n* = 6; *t*-test *p* < 0.05, Table 2).

**Table 2:** The table summarizes the effects of acute and chronic treatment of every single compound on corticostriatal synaptic plasticity.

**Drug**	**Administration**	**Rescue LTD**	**Rescue LTP**
Beta-carotene	Acute	NO	NO
	Chronic		
Lutein	Acute	NO	NO
	Chronic		
Lycopene	Acute	NO	NO
	Chronic		
Folic acid	Acute	NO	NO
	Chronic		
Vitamin A	Acute	NO	NO
	Chronic		
Vitamin C	Acute	NO	NO
	Chronic		
Vitamin K2	Acute	NO	NO
	Chronic		
Vitamin K1	Acute	NO	NO
	Chronic		
Alpha-tocopherol (Vitamin E)	Acute	NO	NO
	Chronic	**YES**	**YES**
Trolox (vitamin E)	Acute	**YES**	**YES**
	Chronic	**YES**	**YES**

*Only alpha-tocopherol and Trolox resulted effective. Bold highlights drugs restoring synaptic plasticity*.

## Discussion

In this study, both the clinical retrospective analysis and our electrophysiological experiments demonstrate that Vitamin E might exert potential beneficial effects in PD.

The case-control analysis showed that dietary VEI is higher in healthy subjects than age/sex matched PD patients. Such a reduced intake in PD patients might suggest a lack of its putative protective action, independently from age and gender. Although consistent with other data from larger and prospective cohorts (17, 18), a number of limitations should be considered in the interpretation of the result, such as the sample size, the recall bias, and the absence of accurate measurement for the dietary intake. In fact, VEI was just approximately estimated by a retrospective *ad hoc* questionnaire, scoring how frequently the standard portions of aliments with Vitamin E higher content were assumed in the last year, and not precisely quantified. However, to prevent confounding factors due to the occurrence of the disease, we excluded from the study patients with alimentary restrictions (e.g., dysphagia), dementia or any concomitant condition affecting feeding behavior or intestinal absorption. Moreover, the homogeneous distribution of demographic features in the study population and the statistical methodology might have limited the influence of other potential confounding factors (e.g., sex/age-based dietary choices, sex/age–dependent differences in internal metabolism). In addition to confirm previous findings (17, 18), here we noticed that VEI did not correlate with severity of PD, assessed by H&Y score. Actually, the sample size and the exclusion from the model of other clinical determinants (e.g., disease duration, therapy) might represent a bias; indeed, it is possible that both levodopa dose and disease duration, which are usually higher in more advanced patients, may affect vitamin absorption (37), causing some deficiency unrelated to the alimentary habits. Also the occurrence and severity of constipation, which in turn might influence dietary choices, pharmacotherapy and intestinal function (38), has not been addressed in the study. Therefore, caution is required in the interpretation of our preliminary results. To this regard, it should be mentioned that other authors, also by utilizing recall-based questionnaires, excluded significant associations between alimentary VEI and PD (39, 40); these studies differed in sample size, but were performed out of Mediterranean area. Hence, we should consider regional diet as a further potential confounding factor. Certainly, prospective cohort studies, eventually supported by direct vitamin dosage, are necessary to assess the weight of VEI in PD pathogenesis.

The protective action of Vitamin E on PD has been further explored by using an experimental model of preclinical PD. *PINK1*^−/−^ mice indeed exhibit the disruption of bidirectional plasticity at corticostriatal synapses, even in the absence of overt neurodegeneration. Several studies indicate this model as representative of a critical time-window of the disease in which a specific intervention may revert the pathophysiological cascade leading to symptoms onset, being thus appropriate to test the efficacy of disease-modifying strategies (3, 14–16). Our experiments show that the administration of Vitamin E (alpha-tocopherol and Trolox), but not other dietary antioxidant compounds (beta-carotene, lycopene, lutein, folic acid, ascorbic acid/Vitamin C, retinol/Vitamin A, Vitamin K1/phylloquinone, and Vitamin K2/menaquinones), was able to revert synaptic plasticity abnormalities in *PINK1*^−/−^ mice. *PINK1* haploinsufficiency precipitates mitochondrial functioning, impairing mitophagy, and above all, energy production under increased demand (41). This, in turn, accounts for the reduced synaptic vesicle release at dopaminergic terminals and the subsequent breakdown of corticostriatal synaptic plasticity (9, 14, 16). It is thus conceivable that Vitamin E, unlike other vitamins, specifically rescues striatal homeostasis and neurotransmission in *PINK1*^−/−^ mice, by enhancing mitochondrial metabolism (42, 43) and energy-dependent processes. Indeed, it has been recently demonstrated that Vitamin E, but not other antioxidants, such as Vitamin C, fully rescued longevity in a short-lived *Candida elegans gas-1(fc21)* model of respiratory chain complex I defect (44). Moreover, in other experimental models, Vitamin E resulted to be able to activate cellular pathways involved in antioxidant, detoxifying, and anti-inflammatory responses and to promote bioenergy at mitochondrial level (45, 46). Regarding *PINK1*^−/−^ rodents model, Shim and colleagues demonstrated that Trolox dramatically improved mitochondrial metabolism in PINK1-deficient dopaminergic cells, by increasing complex I and complex IV's activity (47). Because neurotransmitter release depends on mitochondrial bioenergetics (48), we hypothesize a recovery of a physiological dopaminergic transmission with the subsequent rescue of corticostriatal plasticity. However, a specific set of experiments is required to assess this issue in *PINK1*^−/−^ mice. Yet, the complex interactions between PINK1-mediated mitochondrial activities and Vitamin E-induced cellular reactions (49, 50) could then explain the inefficacy of other proved alimentary antioxidants in restoring corticostriatal synaptic plasticity.

Since mitochondrial dysfunction is critical also in pathogenesis of idiopathic PD (41, 51, 52), such a mechanism may justify a beneficial action of higher dietary VEI on PD in humans. Furthermore, Vitamin E seems to intervene on other pathogenic pathways of PD, such as lysosome metabolism (53), expanding potential restorative effects. Definitely, larger studies are mandatory to validate this hypothesis.

Regardless the limitations, our findings suggest a potential protective action of a Vitamin E rich diet. These data may indicate that Vitamin E represents a potential therapeutic target for disease-modifying treatments in PD. Therefore, diets including Vitamin E rich aliments could be an immediate option to reduce the risk of PD and other neurodegenerative diseases (54), although specific confirmatory trials are necessary.

## Data Availability

The datasets generated for this study are available on request to the corresponding author.

## Author Contributions

TS, GM, and AP conceived the study and wrote the manuscript. GD, VC, DF, and MA collected clinical data. GM and PI performed the experiments. TS and MP performed statistical analysis. NM, MP, and PS contributed to interpretation of results and edited the manuscript.

### Conflict of Interest Statement

The authors declare that the research was conducted in the absence of any commercial or financial relationships that could be construed as a potential conflict of interest.
